# The Challenging Case Conference: A Gamified Approach to Clinical Reasoning in the Video Conference Era

**DOI:** 10.5811/westjem.2020.12.49133

**Published:** 2020-12-23

**Authors:** Scott Kobner, Molly Grassini, Nhu-Nguyen Le, Jeff Riddell

**Affiliations:** LAC+USC Medical Center, Keck School of Medicine, University of Southern California, Department of Emergency Medicine, Los Angeles, California

## Abstract

The development of clinical reasoning abilities is a core competency of emergency medicine (EM) resident education and has historically been accomplished through case conferences and clinical learning. The advent of the SARS-CoV-2 pandemic has fundamentally changed these traditional learning opportunities by causing a nationwide reliance on virtual education environments and reducing the clinical diversity of cases encountered by EM trainees.

We propose an innovative case conference that combines low-fidelity simulation with elements of gamification to foster the development of clinical reasoning skills and increase engagement among trainees during a virtual conference. After a team of residents submits a real clinical case that challenged their clinical reasoning abilities, a different team of residents “plays” through a gamified, simulated version of the case live on a video conference call. The case concludes with a facilitated debriefing led by a simulation-trained faculty, where both the resident teams and live virtual audience discuss the challenges of the case. Participants described how the Challenging Case Conference improved their perceptions of their clinical reasoning skills. Audience members reported increased engagement compared to traditional conferences. Participants also reported an unexpected, destigmatizing effect on the discussion of medical errors produced by this exercise. Residency programs could consider implementing a similar case conference as a component of their conference curriculum.

## BACKGROUND

Physicians commonly learn clinical reasoning through “whole-case” format conferences in which a presenter chronologizes a patient’s presentation, diagnosis, and treatment. However, these conferences may lack characteristics that improve the acquisition of clinical reasoning abilities, such as active learner involvement, uncertainty of clinical practice, and multiple diagnostic or therapeutic choices.^1^–^4^

Alternatives to traditional case conferences may rely on group gatherings that are no longer possible in the era of SARS-CoV-2 (COVID-19).^2^,^4^–^6^ Furthermore, emergency departments nationwide are reporting a decline in the number and diversity of non-COVID-19 cases, increasing trainee’s reliance on supplemented clinical reasoning experiences to meet Accreditation Council for Graduate Medical Education core competencies.^7^,^8^ Teaching clinical reasoning to trainees who are spatially distant can be challenging because audience engagement is difficult during video conferencing, active participation is limited, and financial and technological barriers make virtual simulation difficult.^9^

Gamification—the application of characteristics of games for health professions education—is a modern teaching modality that provides learners with active learning opportunities and improves clinical problem-solving.^10^,^11^ Despite similarities with commercial, tabletop role-playing games, gamification has not been widely adopted for teaching clinical reasoning in low-fidelity simulation settings. Here we provide a description of a novel case conference format that uses the gamification of low-fidelity simulation to teach clinical reasoning skills to a large audience of virtual participants.

## OBJECTIVES

Broadly, we sought to develop a curriculum for the virtual conference format that would foster development of clinical reasoning skills. By gamifying serial-cue, low-fidelity simulations we additionally aimed to improve engagement in clinical case conferences for a spatially distant audience of learners during our weekly residency conference.

## CURRICULUM DESIGN

The Challenging Case Conference consists of three parts: a standardized case submission; a live tabletop simulation; and a subsequent debrief discussion with virtual facilitation.

Prior to the conference, a team of residents submits a real case that challenged their clinical reasoning skills. Case submissions include all relevant diagnostic results, and residents are encouraged to highlight several elements of the case that challenged their clinical reasoning abilities. This submission also includes a brief case conclusion that summarizes their experiences with the case. All clinical data is reviewed by the case facilitator to ensure all patient identifiers have been removed prior to inclusion in the conference.

Next, a different team of 3–5 residents, with no knowledge of the case, “plays” through a simulated tabletop version of the case live on a video conference call during our weekly resident didactics. This team was comprised of members across all four postgraduate years, meeting in person and in virtual attendance. Akin to a mock oral boards case, this case flow is facilitated by a chief resident familiar with serial-cue tabletop simulation and gaming techniques.

**Figure 1:**
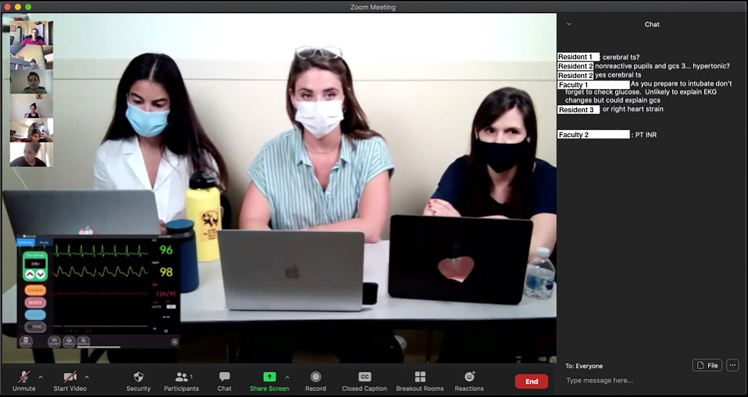
Screenshot of the Zoom application during the case simulation. Resident participants (center screen) play through a simulated case on a live video broadcast, with real-time simulated clinical data overlayed on the video stream (bottom left corner) for audience members (top left) to view. A scrolling, real-time chat feed (right) is visible to audience and participants alike.

Whenever participants ask to perform interventions during the case, they are instructed to roll a set of dice. On a roll of 10–12, the action is successful (the patient gets intubated); on a roll of 7–9, success comes with an unexpected consequence (intravenous access was obtained, but only with a small bore catheter); on a roll of 1–6, the action is unsuccessful (the patient became hypotensive). Vital signs are provided to the team and virtual audience through Simpl (a commercially available simulated cardiac monitor, www.simplsim.com). All de-identified lab and imaging results are housed in a Dropbox (www.dropbox.com) folder. As team-members ask for diagnostic studies during the play of the case, a facilitator puts a hyperlink to the Dropbox file correlating with their request (chest radiograph, urinalysis, etc.) in the virtual chat room, making the data available to both team members and the audience (Figure 1).

The resident team working through the clinical case can see the virtual chatroom, where other trainees and faculty can remotely comment on the case as it unfolds. At the conclusion of the case, simulation-trained faculty lead a debriefing with the team that submitted the case and the simulation participants. The two teams discuss the various challenges present in the case and engage in a real-time discussion with the virtual audience.

This educational methodology was chosen to combine the strengths of gamification, serial-cue case discussions, and low-fidelity simulation while minimizing the known obstacles imposed by the virtual environment. The moment-to-moment uncertainty that is theorized to improve clinical reasoning skills in a serial-cue case approach is naturally complemented by the gamified uncertainty of dice probabilities.^3^,^7^

## IMPACT/EFFECTIVENESS

During a recent conference, this exercise took 43 minutes, involved a virtual audience of 89 trainees and faculty, and had 52 distinct comments in the chatroom. Chat comments were largely real-time clinical reasoning pearls, questions about the case, and suggestions to participants. Following the case debriefing, 11 minutes of spontaneous faculty discussion ensued, which covered themes ranging from diagnostic decision- making and airway management to systems-level patient safety issues and foundational medical knowledge.

We interviewed a convenience sample of volunteer attendees directly following this conference, which included three simulation participants, five resident audience members, and three residency program administrators. Semi-structured interviews were conducted with a standardized set of questions (Appendix 1) designed to explore perceptions of the curriculum from multiple sources. Transcribed responses underwent a basic thematic analysis to reveal several key trends. All simulation participants felt that the tabletop simulation improved their clinical reasoning ability in ways that mimicked real clinical encounters. They reported that the dice element both added a level of unpredictability, which helped model the actual practice of emergency medicine (EM) and also added a level of excitement absent from typical mock oral boards-type tabletop simulations. All three residents initially endorsed anxiety participating in front of a live virtual audience, but agreed that this anxiety dissipated during the exercise. Interestingly, all participants described a normalization phenomenon, wherein openly acknowledging challenges with the clinical reasoning in the simulation and real case produced an honest, stigma-free discussion of medical errors.

Audience members agreed that they were more engaged throughout the case simulation than during traditional case conferences due to the format and gamification. Two junior trainees reported that the gamification allowed them to witness participants work through uncertainty in real time, which informed their developing practice. Residency program administrators noted increased faculty engagement and discussion when compared to historical case conferences. Finally, interviewees suggested using an alternative to Dropbox to view diagnostic results to limit interruptions in the case flow and to use more gamification throughout the simulation.

## CONCLUSION

The Challenging Case Conference uses elements of low-fidelity simulation and serious games to increase perceptions of clinical reasoning skills for a virtual audience of EM trainees, while also increasing perceptions of virtual participation.

## Supplementary Information
