# Supplementary material for: The Challenging Case Conference: A Gamified Approach to Clinical Reasoning in the Video Conference Era
Source: West J Emerg Med. 2020 Dec 23;22(1):136–8. doi: 10.5811/westjem.2020.12.49133 (PMC7806328; doi:10.5811/westjem.2020.12.49133)
Supplement: Supplementary file 1 [file wjem-22-136-s001.docx]

**Appendix 1: Interview Questions**

1. What was your overall impression of the Challenging Case Conference?
2. How did the Challenging Case Conference compare to other case discussions you have attended?
3. Did the Challenging Case Conference contribute to your clinical reasoning? How so?
4. What was your overall impression of the game elements (dice rolling) of the conference?
5. Would you be willing to participate in a Challenging Case Conference? Why or why not?
6. Did you find the Challenging Case Conference engaging when compared to other case-based didactics?
7. Where there any technical challenges to viewing or participating in the Challenging Case Conference?
8. What about the Challenging Case Conference worked well?
9. What about the Challenging Case Conference could be improved?
10. Do you have any other thoughts about the Challenging Case Conference?
